# Influence of prefoldin subunit 4 on the tolerance of *Kluyveromyces marxianus* to lignocellulosic biomass-derived inhibitors

**DOI:** 10.1186/s12934-021-01715-y

**Published:** 2021-12-14

**Authors:** Nini Zhang, Yingying Shang, Feier Wang, Dongmei Wang, Jiong Hong

**Affiliations:** 1 School of Life Sciences, University of Science and Technology of China, Hefei, 230027 Anhui People’s Republic of China; 2 Hefei National Laboratory for Physical Science at the Microscale, Hefei, Anhui 230026 People’s Republic of China; 3 Biomedical Sciences and Health Laboratory of Anhui Province, University of Science & Technology of China, Hefei, 230027 China

**Keywords:** Prefoldin, *KmPFD4*, Lignocellulosic biomass, Inhibitor tolerance, *Kluyveromyces marxianus*

## Abstract

**Background:**

*Kluyveromyces marxianus* is a potentially excellent host for microbial cell factories using lignocellulosic biomass, due to its thermotolerance, high growth rate, and wide substrate spectrum. However, its tolerance to inhibitors derived from lignocellulosic biomass pretreatment needs to be improved. The prefoldin complex assists the folding of cytoskeleton which relates to the stress tolerance, moreover, several subunits of prefoldin have been verified to be involved in gene expression regulation. With the presence of inhibitors, the expression of a gene coding the subunit 4 of prefoldin (*KmPFD4*), a possible transcription factor, was significantly changed. Therefore, *KmPFD4* was selected to evaluate its functions in inhibitors tolerance.

**Results:**

In this study, the disruption of the prefoldin subunit 4 gene (*KmPFD4*) led to increased concentration of intracellular reactive oxygen species (ROS) and disturbed the assembly of actin and tubulin in the presence of inhibitors, resulting in reduced inhibitor tolerance. Nuclear localization of KmPFD4 indicated that it could regulate gene expression. Transcriptomic analysis showed that upregulated gene expression related to ROS elimination, ATP production, and NAD^+^ synthesis, which is a response to the presence of inhibitors, disappeared in *KmPFD4*-disrupted cells. Thus, *KmPFD4* impacts inhibitor tolerance by maintaining integration of the cytoskeleton and directly or indirectly affecting the expression of genes in response to inhibitors. Finally, overexpression of *KmPFD4* enhanced ethanol fermentation with a 46.27% improvement in productivity in presence of the inhibitors.

**Conclusion:**

This study demonstrated that *KmPFD4* plays a positive role in the inhibitor tolerance and can be applied for the development of inhibitor-tolerant platform strains.

**Supplementary Information:**

The online version contains supplementary material available at 10.1186/s12934-021-01715-y.

## Background

Global energy consumption could reach more than 10^6^ quadrillion British thermal unit by 2050 [1]. With increased demand for energy and chemicals, more fossil fuels are being consumed. Not only have the limited crude oil and coal supplies become depleted, but excessive consumption of fossil fuels has led to global warming due to the emission of large amounts of greenhouse gases and organic pollutants. Therefore, more attention is being paid to the development of biofuel and green chemicals. Lignocellulosic biomass is an important feedstock for bioethanol and other chemicals because it is renewable and sustainable. In general, more than 220 billion tons of lignocellulosic biomass is produced annually [2]. Large amounts of lignocellulosic biomass make it possible, at least partly, to substitute fossil fuel energy and resources. Lignocellulosic biomasses are more attractive than corn and sugarcane because they do not compete with food [3].

Pretreatment is required to improve the efficiency of lignocellulosic biomass hydrolysis and the fermentable sugar yield. Lignocellulosic biomass is mainly composed of cellulose, hemicellulose, and lignin with a ratio in the range of 30–55 wt%, 15–40 wt%, and 10–35 wt%, respectively [1]. Glucose, xylose, arabinose, and other sugars can be released from lignocellulosic biomass through enzymatic hydrolysis [4]. However, crystallized cellulose, hemicellulose, and highly polymerized phenolic lignin lead to difficulty in lignocellulosic biomass hydrolysis [5]. Therefore, pretreatment, which can destroy the structure of lignocellulosic biomasses, improves accessibility and enables easy hydrolysis. During pretreatment, inhibitors which inhibit the growth and the fermentation ability of microbes are produced. These inhibitors are mainly weak acids, furan compounds, and phenolic chemicals. Weak acids include formic acid, acetic acid, and levulinic acid. Furfuran compounds generally refer to furfural and 5-hydroxymethyl-2-furaldehyde (HMF), whereas phenolic chemicals are mainly the compounds from the degradation of lignin and other aromatic compounds obtained from biomass [4, 6].

Furfural and 5-hydroxymethylfurfural (HMF) can negatively affect the microbial fermentation by inhibiting cell growth and sugar uptake rate, subsequently reducing ethanol production rate. Furfuran compounds also have negative effects on metabolisms, cell wall formation, and DNA, RNA and/or protein synthesis [7]. The primary carbon catabolism enzymes including acetaldehyde dehydrogenase, alcohol dehydrogenase, aldehyde dehydrogenase, glyceraldehydre-3-phosphate dehydrogenase, and pyruvate dehydrogenase are also repressed by furfuran compounds [6–9]. Inhibition mechanisms of phenolic compounds on eukaryotic microorganisms have not yet been completely elucidated [8]. Phenolic compounds lead to a loss of integrity in cell membrane membranes, thereby affecting their ability to serve as selective barriers and enzyme matrices. Consequently, phenolic compounds reduce cell growth, sugar assimilation, and fermentation [6]. Phenolic compounds are also able to penetrate cell membranes and damage internal structures, as well as causing changes in the morphology of cells [6]. Weak acids reduce the cytosolic pH [10]. The protons, then, are pumped out of the cell through the plasma membrane ATPase with ATP hydrolysis. Consequently, less ATP is available for biomass formation. With the presence of higher concentrations, the ATP requirements increased and cells cytosol is acidified [11]. Furfuran compounds, acetic acid, and phenolic compounds lead to accumulation of reactive oxygen species (ROS) in yeast, and subsequently cause cellular damage including damage to mitochondria and vacuole membranes, the actin cytoskeleton and nuclear chromatin [12, 13].

Yeast cells metabolize or pump out the inhibitors to tolerate the inhibitors. Phenolic compounds in the inhibitors can be converted to less toxic compounds. For instance, coniferyl aldehyde is reduced to coniferyl alcohol and dihydroconiferyl alcohol. Furfurals are metabolized into less toxic acid or alcohol forms using NAD(P)H as cofactor [11]. The furfurals and phenolic compounds were also proved to be reduced to corresponding alcohol in *K. marxianus* by Oliva group and in our previous study [14, 15]. However, when the concentration of inhibitors is high, the NAD(P)H and ATP for the inhibitors conversion, ROS elimination and inhibitors pumping out are not enough, the growth of the yeast is hindered, even death under severe conditions.

Improvement of inhibitor tolerance of microbes could enhance fermentation and reduce the cost of inhibitors removal. The lignocellulosic biomass-derived inhibitors can be removed through physical, chemical, or biological methods [16]. However, inhibitors removal by physical or chemical methods incurs greater costs in industrial production. Many microorganisms can degrade the lignocellulosic biomass-derived inhibitors, although this procedure is generally time-consuming. The construction of genetically engineered microbes with improved inhibitor tolerance is another important approach that can prevent or reduce the cost of inhibitors removal [8].

Studies regarding the inhibitor tolerance mechanism of *Kluyveromyces marxianus* are necessary and can improve its inhibitor tolerance. *K. marxianus* is a nontraditional yeast and generally regarded as safe [17]. Its thermotolerance enables fermentation at elevated temperatures, which can be used in the tropic region and high temperature season with reduced contamination [18–21]. *K. marxianus* is considered to be the fastest-growing eukaryote with highest possible replication rate [22] and the high growth rate of *K. marxianus* could improve the production rate. The pentose utilization ability of *K. marxianus* is attractive for fermentation with lignocellulosic biomass hydrolysate. The combination of thermotolerance and wide substrate spectrum makes *K. marxianus* suitable for the simultaneous saccharification and fermentation or simultaneous saccharification and co-fermentation of lignocellulosic biomass [15, 23–25]. *K. marxianus* has many advantages, and its tolerance to single kind inhibitor is higher than that of the widely used *Saccharomyces cerevisiae* [14, 26–28]. However, it is still necessary to improve the tolerance of *K. marxianus* to multiple inhibitors.

Prefoldin is a co-chaperone that is widely present in archaea and eukaryotes, and facilitates the supply of unfolded or partially folded substrates to class II chaperonin chaperonin-containing TCP-1. In canonical prefoldin, four β-type subunits (eukaryotic PFD1, 2, 4, and 6) form two dimers onto two subunits of α type (PFD3 and 5) [29]. The canonical prefoldin complex helps actin and tubulin assembly and the folding of other proteins with chaperones [30]. Prefoldin is also a gene regulator [29]. The increased levels of subunit PFD1 represses cyclin A expression by directly interacting with its promoter at the transcriptional start site [31]. PFD3 influences the action of the viral HBx protein as a transcriptional coactivator [32]. PFD4 is reported with possible transcription factor activity in human cells [33]. The subunit PFD5 acts as a co-repressor of the E-box-dependent transactivation activity of c-Myc [34]. Subunits PFD5 and PFD6 play a relevant role in gene expression in relation to DELLA nuclear factors, which are known to regulate the expression of a large set of genes in plants[35]. Finally, yeast prefoldin subunits PFD1, PFD2, PFD5 and PFD6 bind yeast chromatin in a transcription-dependent manner following a profile that parallels the phosphorylation of the Ser2 residues of RNA pol II C-terminal domain, and play a positive role in transcription elongation [36]. However, the functions of prefoldin in the tolerance to lignocellulosic biomass-derived inhibitors have not been reported.

Our transcriptomic analysis illustrated that, with the presence of multiple lignocellulosic biomass-derived inhibitors, the subunits of prefoldin in *K. marxianus* except PFD5 are down-regulated (Additional file 1). The real-time PCR (RT-PCR) results indicated that compared to the expression at exponential phase, the expression of most prefoldin subunits increased at stationary phase except the *KmPFD1*expression with no inhibitor and *KmPFD4* expression with inhibitors (Additional file 2: Fig. S1). Since PFD4 has not been as well studied as other subunits, *KmPFD4* in *K. marxianus* was disrupted or overexpressed to evaluate its effect on the tolerance to inhibitors. The effect of the disruption of *KmPFD4* on actin and tubulin, the intracellular location of KmPFD4, intracellular ROS, and the transcriptome were determined to elucidate the mechanism of inhibitor tolerance. Furthermore, the effect of disruption on the tolerance towards other kinds of stress, such as salt tolerance, temperature, etc.*,* was evaluated as well. Finally, the effect of overexpressing *KmPFD4* in ethanol fermentation in the presence of inhibitors was also evaluated.

## Materials and methods

### Reagents and original strain

D-glucose, D-xylose, and yeast nitrogen base without amino acids (YNB) were obtained from Sangon Biotech Co. (Shanghai, China). Restriction endonucleases were purchased from Thermo Fisher Scientific (West Palm Beach, Florida, USA). Yeast extract, tryptone, and bacteriological peptone were acquired from Oxoid (Oxoid Ltd., Basingstoke, Hampshire, UK). *K. marxianus* YHJ010, a *trp1*, *leu2*, and *ura3* auxotroph derived from *K. marxianus* NBRC 1777, was used as the original strain [37]. Synthetic dropout (SD) medium (6.7 g/L YNB, 20 g/L glucose) was used for transformant selection. Yeast extract/peptone-dextrose (YPD) medium (10 g/L yeast extract, 20 g/L bacteriological peptone, and 20 g/L D-glucose) was used for the cultivation of *K. marxianus* strains. To prepare solid plates of each medium, 1.5% (w/v) agar was added. *Escherichia coli* DH5α was used as the host for gene cloning and vector construction,and was cultivated in lysogeny broth medium supplemented with 100 μg/mL ampicillin.

### Plasmids construction

DNA fragment of prefoldin subunit 4 (*KmPFD4*, GenBank accession No. BAP73153, locus_tag: KMAR_60414) was amplified from the genomic DNA of *K. marxianus* YHJ010 with primers KmPFD4-F and KmPFD4-R (Additional file 2: Table S1). The amplified fragment was inserted into pGEM-T easy (Promega Corporation, Madison, WI, USA), and the resultant plasmid was pSY001 (Table 1).

**Table 1:** Plasmids used in this study

Plasmids	Description	References
YEGAP	*Amp*^*R*^, *ScTRP1,* P_*ScGAPDH*_*,* T_*ScGAPDH*_	[37]
YEUGAP	*Amp*^*R*^, *ScURA3*, P_*ScGAPDH*_, T_*ScGAPDH*_	[17]
YEUKmPGK	*Amp*^*R*^, *ScURA3,* P_*KmPGK*_*,* T_*ScGAPDH*_	[38]
pGEM-T Easy	*Amp*^*R*^	Promega
pGEM-T-△*ScURA3*	*Amp*^*R*^, nonfunctional *ScURA3*	[18]
pPCG	*Zeocin*^*R*^, CBM-EGFP- pPICZ αA	[39]
pSY001	*Amp*^*R*^, *KmPFD4*-pGEM-T Easy vector AmpR, KmPFD4-T vector	This study
pSY002	*Amp*^*R*^, *KmPFD4* inserted with *ScURA3*	This study
pSY003	*Amp*^*R*^, *ScTRP1*, P_*ScGAPDH*_- *KmPFD4* -T_*ScGAPDH*_	This study
pSY004	*Amp*^*R*^, *ScURA3*, P_*ScGAPDH*_-*EGFP*- T_*ScGAPDH*_	This study
pSY005	*Amp*^*R*^, *ScURA3*, P_*ScGAPDH*_- *KmPFD4* -*EGFP*-T_*ScGAPDH*_	This study

**Fig. 1:**
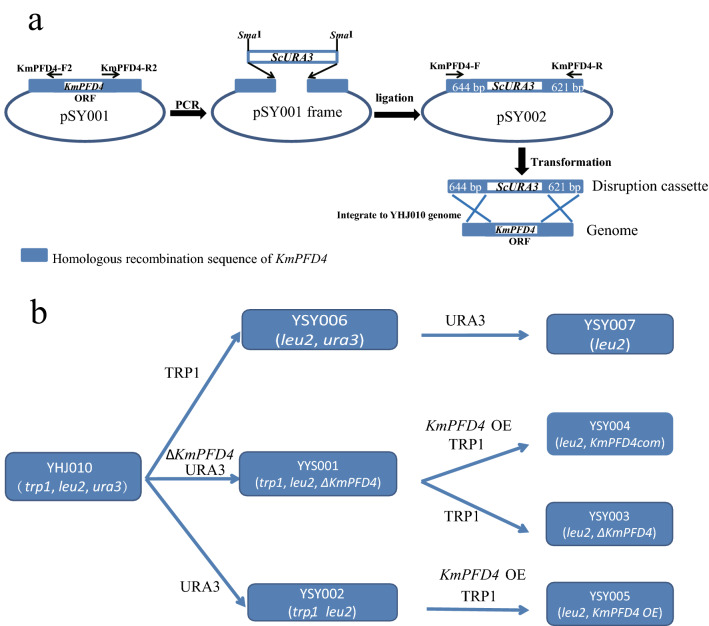
Schematic diagram for the construction of strains. **a** Construction of the *KmPFD4* gene disruption cassette and gene disruption, **b** The strain construction procedure. Δ: disruption, OE: overexpression

The *KmPFD4* disruption cassette was constructed in plasmid pSY002. The frame of plasmid pSY001 and part of *KmPFD4* were amplified with primers KmPFD4-F2 and KmPFD4-R2 (Additional file 2: Table S1; Fig. 1). After the *ScURA3* expression cassette including the original promoter, ORF, and terminator of *ScURA3* was amplified from YEUGAP [17] with primers ScURA3-SmaI-F and ScURA3-SmaI-R (Additional file 2: Table S1), it was ligated with the amplified pSY001 frame (Fig. 1). The obtained plasmid was pSY002 (Table 1). The *KmPDF4* disruption cassette in pSY002 included 644 bp upstream and 621 bp downstream homologous recombination sequences of *KmPDF4* and *ScURA3* expression cassette (Fig. 1).

The *KmPFD4* overexpression plasmid was constructed as follows: the open reading frame (ORF) of *KmPFD4* was amplified from pYS001 with primers KmPFD4-EcoRI-F and KmPFD4-NotI-R (Additional file 2: Table S1) and inserted into the YEGAP plasmid [17] at *Eco*RI and *Not*I sites. The resultant plasmid was named pSY003 (Table 1).

Two plasmids were constructed for the intracellular localization of KmPFD4. The enhanced green florescent protein (*EGFP*) gene was amplified from pPCG [39] with the primers EGFP-EcoRI-F and EGFP-NotI-R and inserted into YEGAP between *Eco*RI and *Not*I sites. The resultant plasmid pSY004 (Table 1) was used to construct a control strain for *EGFP* expression in *K. marxianus*. The ORF of *KmPFD4* was amplified from pSY001 with primers KmPFD4-EcoRI-F and KmPFD4-fusion-R. At the same time, the *EGFP* gene was amplified from pPCG with the primers EGFP-fusion-F and EGFP-NotI-R. The resultant *KmPFD4* and *EGFP* DNA fragments were fused together with primers KmPFD4-EcoRI-F and EGFP-NotI-R through overlap extension PCR. The fused *KmPFD4-EGFP* fragment was digested with *Eco*RI and *Not*I and inserted into YEGAP. The resultant plasmid was pSY005 (Table 1).

### Strains construction

The *KmPFD4* disrupted strain was constructed with YHJ010. The gene disruption cassette was amplified from pSY002 using primers KmPFD4-F and KmPFD4-R and transformed into YHJ010 by the lithium acetate method [40]. After screening on SD medium supplemented with leucine and tryptophan, the positive clones were confirmed by PCR with genomic DNA as the template. The obtained *KmPFD4*-disrupted strain was named YSY001 (Table 2). Subsequently, the empty plasmids YEGAP or pSY003 were transformed into YSY001, and the obtained strains were YSY003 and YSY004, respectively (Table 2). YSY003 was a *KmPFD4*-disrupted *URA3* and *TRP1* auxotroph-complemented strain. YSY004 was a retro-complementary strain of *KmPFD4* disruption (Table 2). To confirm if more *KmPFD4* could enhance the inhibitor tolerance of *K. marxianus*, the *KmPFD4* was overexpressed with strong promoter (TDH3).

**Table 2:** Strains used in this study

Strains	Description	References
YHJ010	*K. marxianus* NBRC1777*, ΔKmURA3::Kanr, ΔKmLEU2::hisG, △KmTRP1::hisG*	[37]
YSY001	*K. marxianus,* YHJ010*, ΔKmPFD4::ScURA3*	This study
YSY002	*K. marxianus,* YHJ010*, ScURA3*	This study
YSY003	*K. marxianus* YSY001*, ScTRP1*	This study
YSY004	*K. marxianus* YSY001*, ScTRP1, KmPFD4*	This study
YSY005	*K. marxianus* YSY002*, ScTRP1, KmPFD4*	This study
YSY006	*K. marxianus* YHJ010*, ScTRP1*	This study
YSY007	*K. marxianus* YSY006*, ScURA3*	This study
YSY008	*K. marxianus* YHJ010*, EGFP*	This study
YSY009	*K. marxianus* YHJ010, *KmPFD4-EGFP*	This study

The *ScURA3* expression cassette was amplified from YEUGAP with primers ScURA3-SmaI-F and ScURA3-SmaI-R and transformed into YHJ010. The resultant strain was named YSY002, which is a wild-type *KmPFD4* and selection marker-complemented strain. YSY002 was then transformed with the pSY003 and the resultant YSY005 was the *KmPFD4* overexpression strain.

YHJ010 was transformed with the plasmid YEGAP [37] and *ScURA3* expression cassette in turn to obtain YSY006 and YSY007.YSY007 was the *URA3* and *TRP1* auxotroph marker-complemented strain, which was used as a wild-type control.

pSY004 or pSY005 was linearized and transformed into YHJ010. YSY008 and YSY009 were obtained and used for intracellular localization of KmPFD4. These strains expressed *EGFP* and *KmPFD4-EGFP*, respectively.

### Growth assay of *KmPFD4* disruption or overexpression on inhibitors or other stress tolerance

YSY003, YSY004, YSY005, and YSY007 were inoculated into 5 mL of YPD and cultivated overnight at 42 °C with 250 rpm shaking. The overnight cultures were inoculated into 30 mL of YPD medium (pH 6.0) containing no inhibitor, various inhibitors, 20 g/L ethanol, 180 g/L glucose, or 0.5 M NaCl with a starting OD_600_ = 0.5 in a 250 mL flask. Then, they were cultivated at 42 °C with shaking at 250 rpm. The OD_600_ was monitored during growth. Cultivation at 45 °C was used as temperature stress condition. The inhibitors used included mixed inhibitors, 20 g/L acetate sodium, 2.0 g/L furfurals (1.0 g/L furfural and 1.0 g/L HMF), or 1.3 g/L phenols (4-hydroxybenzaldehyde, syringaldehyde, catechol, and vanillin with 0.325 g/L of each compound). The mixed inhibitors contained 2.0 g/L acetate sodium, 0.5 g/L furfural, 0.5 g/L HMF, 0.05 g/L 4-hydroxybenzaldehyde, 0.05 g/L syringaldehyde, 0.05 g/L catechol, and 0.05 g/L vanillin.

### Intracellular ROS assay

The intracellular concentration of ROS was determined by 2,7-dichlorodihydrofluorescein diacetate (DCFH-DA) staining. Strains YSY003, YSY004, YSY005, and YSY007 were inoculated into 30 mL of YPD medium with starting OD_600_ = 0.5 and cultivated at 42 °C with 250 rpm shaking. When the cell density reached 6 (OD_600_), the cells were recovered by centrifugation at 5000×*g* and resuspended in YPD (pH 6.0) containing mixed inhibitors and continuously cultured for 2 h at 42 °C with 250 rpm shaking. The mixed inhibitors contained 5.3 g/L acetate sodium, 1.3 g/L furfural, 1.3 g/L HMF, 0.125 g/L 4-hydroxybenzaldehyde, 0.125 g/L syringaldehyde, 0.125 g/L catechol, and 0.125 g/L vanillin. The cells were then recovered and stained with DCFH-DA [41]. Fluorescence was detected by using a CLARIOstar multimode microplate reader. The excitation and emission wavelengths were 488 and 525 nm, respectively. The concentrations of inhibitors used in ROS determination were higher than those used in growth curve determination.

### Determine the effect of KmPFD4 to the respiratory efficiency of *K. marxianus*

YSY003 or YSY007 strain was inoculated in 5 mL YPD medium and cultivated at 37 °C overnight. Then, the preculture was diluted 5000-fold with water, and 5 μl cell suspension was inoculated on YPD plate which contained no or mixed inhibitors. The concentration of inhibitors was same as used in growth assay. After cultivation at 37 °C for 18 h, a top agar containing 1% (w/v) agar, 0.5%(w/v) glucose, and 0.005%(w/v)2,3,5-Triphenyltetrazolium chloride (TTC) was overlaid on the plate. Then the plate was incubated at 30 °C for 3 h. The lighter color of the colonies indicated respiratory deficiency [42].

### RNA-seq analysis of the KmPFD4-disrupted strain

After the cells of *K. marxianus* YSY003 or YSY007 were cultivated with YPD (pH 6.0) containing mixed inhibitors and continuously cultured for 2 h at 42 °C with 250 rpm shaking, the cells were then frozen in liquid nitrogen. The inhibitors concentration and culture conditions were the same as described in “Intracellular ROS assay” section. The cells cultivate in the YPD medium (pH6.0) without inhibitors were also collected as control. Total RNA extraction, cDNA library preparation, and RNA-seq analysis performed by Majorbio Co., Ltd (Shanghai, China). The clean reads were mapped to the reference genome of *K. marxianus* NBRC1777 from GenBank (accession No. AP014599-AP014607 using TopHat (http://ccb.jhu.edu/software/tophat/index.shtml).

For gene function annotations, obtained unigene sequences were annotated by searching in various protein databases, including the National Center for Biotechnology Information (NCBI) non-redundant protein (NR) database, the NCBI NR nucleotide sequence (Nt) database, Swiss-Prot, Pfam, Cluster of Orthologous Groups of proteins (COG), Gene Ontology (GO), and Kyoto Encyclopedia of Genes and Genomes (KEGG).

For different gene expression analysis, transcripts per million (TPM) were used as a value of normalized gene expression, and genes were considered differentially expressed in a given library when *p* < 0.001 and a greater than two-fold-change (FC) in expression across libraries was observed using the webtool DEGSeq (https://bioconductor.org/packages/stats/bioc/DEGSeq/). These genes were annotated as differentially expressed genes (DEGs).

### Real-time PCR analysis

Real-time PCR (RT-PCR) was used to analyze the expression level of each subunit of prefoldin and validation of RNA-seq results. Total RNA was isolated using a yeast total RNA extraction kit (Sangon Biotech Co. Shanghai, China). The genomic DNA in isolated RNA was removed by gDNA Eraser (SparkJade Science Co., Ltd., Qingdao, China) and cDNA was synthesized using the SPARKscript II RT Plus Kit (SparkJade Science Co., Ltd., Qingdao, China) [27]. Real-time PCR was conducted on a Roche LightCycler®96 (Roche Molecular Systems, Inc., CA, USA) using the ChamQ Universal SYBR qPCR Master Mix kit (Vazyme Biotech Co.,Ltd, Nanjing, China). The primers for each gene and the *ACT1* internal control are shown in Additional file 2: Table S1. The cycle threshold values (*C*_T_) were determined and the relative fold differences were calculated using the 2^-ΔΔCT^ method [43] with *ACT1* as the endogenous reference gene.

### Evaluation of tolerance to inhibitors and stress by solid medium

The cells of the evaluated strains were cultivated in YPD medium with a starting OD_600_ = 0.5 at 42 °C with 250 rpm shaking until OD_600_ = 6. The cells were recovered by centrifugation at 12,000 × g and resuspended in sterilized water. Then, cells were diluted to OD_600_ = 6 × 10^–1^, 6 × 10^–2^, 6 × 10^–3^, and 6 × 10^–4^ and 3 μL of cells were spotted on the plates. For the inhibitor tolerance, the mixed inhibitors, including 2.0 g/L acetic acid, 0.5 g/L furfural, 0.5 g/L HMF and 0.2 g/L phenols,were added to the YPD (pH 6.0) solid medium. As to the single type of inhibitor treatment, 20 g/L acetic sodium, the furfurals including 0.5 g/L furfural and 0.5 g/L HMF, or the phenols containing 1.3 g/L phenols was added to the YPD (pH 6.0) solid medium. To evaluate the effect on tubulin in the *KmPFD4*-disrupted strain, 7 μg/mL benomyl or 0.7 M NaCl was added to the YPD solid medium.

### Intracellular localization of KmPFD4

To analyze the mechanism of *KmPFD4* in inhibitor tolerance, intracellular localization was determined. YSY008 (EGFP) and YSY009 (KmPFD4-EGFP) were cultivated in YPD or YPD containing mixed inhibitors. YSY008 or YSY009 was cultivated at 42 °C until the OD_600_ reached 6. Then, 500 μL of the cells were recovered by centrifugation at 12,000×*g* and stained with 4,6-diamidino-2-phenylindole (DAPI; Sigma, USA) as previously described (Amberg et al. [44]. The imaging data were collected using a Perkin Elmer Ultraview VoX Spinning Disk Microscope equipped with a Hamamatsu C9100-23B EMCCD camera and a CFI Apochromat TIRF 100 × objective (NA = 1.49). The excitation wavelengths for EGFP and DAPI were 488 and 405 nm, respectively.

### Detecting the effect of KmPFD4 disruption on actin assembly

The effect of *KmPFD4* disruption on actin was detected using the TRITC phalloidin staining. YSY003 (*KmPFD* disrupted) and YSY007 (control) were cultivated in YPD medium at 42 °C with 250 rpm shaking until OD_600_ = 6. Then, the cells were recovered and stained with 200 nM TRITC phalloidin as previously described [45]. The imaging data were collected using a Perkin Elmer Ultraview VoX Spinning Disk Microscope. The excitation wavelength for TRITC phalloidin was 561 nm.

### Evaluating the effect of KmPFD4 overexpression on anaerobic fermentation

YSY005 and YSY007 were cultivated in 5 mL of YPD at 42 °C and shaken at 250 rpm overnight. The precultures were then inoculated into 20 mL of YPD medium containing 100 g/L glucose, 1.9 g/L acetic acid, 0.95 g/L furfurals, and 0.19 g/L phenols (four phenols with the same concentration) in anaerobic bottles. The initial cells density was OD_600_ = 0.3, and the growth (OD_600_), glucose consumption, and ethanol production were measured during the fermentation process.

### Analytical methods

The growth of yeast was examined by measuring the OD_600_. Glucose and ethanol were quantified by high-performance liquid chromatography with a ROA-Organic Acid H^+^ (8%) column (Phenomenex, USA). The mobile phase was 0.0025 M H_2_SO_4_ at a column temperature of 75 °C and a flow-rate of 0.3 mL/min.

### Statistical analysis

All experiments were repeated for three times and the standard error of the mean was marked as error bars in figures.

## Results and discussions

### *KmPFD4* gene cloning

A 1590 bp DNA fragment of *KmPFD4* gene including 600 bp upstream and 600 bp downstream of ORF was amplified from the genomic DNA of *K. marxianus* YHJ010 (Additional file 2: Fig. S2). The ORF encoded a protein of 129 amino acid residues. The amino acid sequence of KmPFD4 was of 87.60%, 69.77%, and 55.38% identity to PFD4 from *K. lactis*, *S. cerevisiae*, and *Candida albicans*, respectively (Additional file 2: Fig. S2).

### *KmPFD4* disruption, complementary, and overexpression strains were obtained

**Fig. 2:**
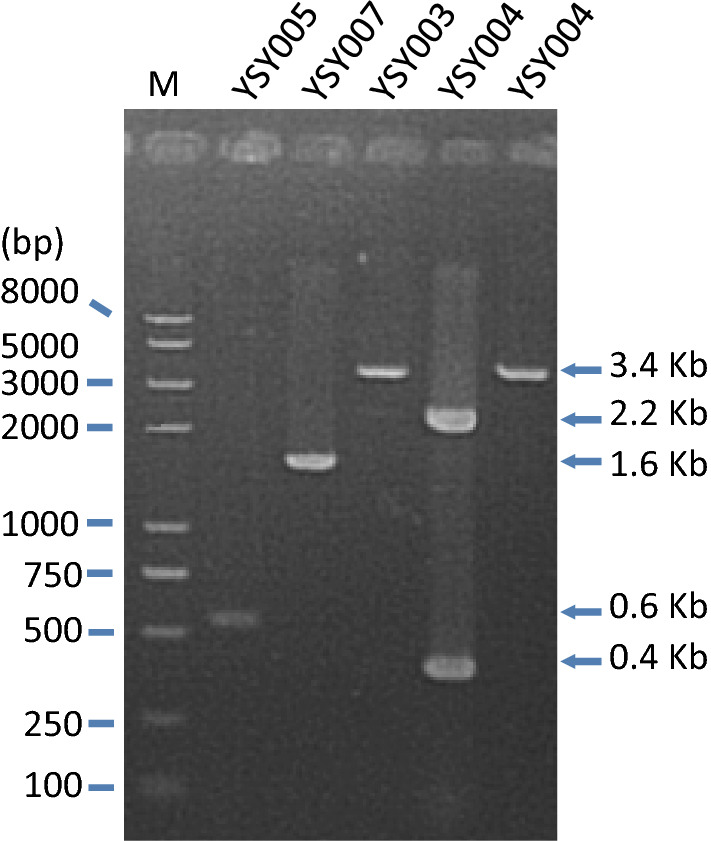
Confirmation of the constructed YSY003 (ΔKmPFD4), YSY004 (complementary strain), YSY005 (*KmPFD4* overexpression), and YSY007 (non-disruption control) through PCR. Their genomic DNA was used as the template

*KmPFD4* disrupted strain YSY003, *KmPFD4* disrupted and retro-complementary strain YSY004, *KmPFD4* overexpression strain YSY005, and *KmPFD4* non-disruption control strain YSY007 were obtained. For a fair comparison in the following investigations, these strains were the same auxotroph (*leu2*). Figure 2 shows the confirmation results of PCR with genomic DNA as the template. The *KmPFD4* in YSY007 (Table 2) was unmodified, and a 1.6 kb DNA fragment was amplified with primers KmPFD4-F and KmPFD4-R (Fig. 2). In YSY003, part of the ORF of *KmPFD4* was substituted with a 2.2 kb DNA (*ScURA3* expression cassette), and only a 3.4 kb DNA portion was amplified with primers KmPFD4-F and KmPFD4-R. If *KmPFD4* was not disrupted, a 1.6 kb DNA was expected to be observed. YSY005 was an overexpression strain of *KmPFD4*. To differentiate the transformed *KmPFD4* and original *KmPFD4*, a primer based on the terminator of the overexpression cassette (TDH3-Ter-R) was used with primer KmPFD4-EcoRI-F to amplify the overexpressed *KmPFD4*. A 0.6 kb fragment, which including 0.4 kb ORF and 0.2 kb terminator, was amplified (Fig. 2). YSY004 was constructed for overexpressing *KmPFD4* in the *KmPFD4* disrupted strain to complement the disruption of *KmPFD4*. Using the primers for the disruption cassette (KmPFD4-F and KmPFD4-R), a 3.4 kb DNA fragment was detected. Using the *KmPFD4* ORF primers KmPFD4-EcoRI-F and KmPFD4-NotI-R, a disrupted *KmPFD4* (2.2 Kb), and a wild-type *KmPFD4* (0.4 Kb) were detected (Fig. 2). The expression of *KmPFD4* in YSY003, YSY004, YSY005, and YSY007 strains were verified through RT-PCR. With the expression of *ACT1* as internal control, the relative expression levels (2^−ΔΔCt^) were 0.00, 0.17, 0.20, and 0.04, respectively (Additional file 2: Fig. S3). The very low expression level of *KmPFD4* in YSY003 indicated that *KmPFD4* was disrupted. And the higher expression level of *KmPFD4* in YSY004 and YSY005 than YSY007 indicated that the *KmPFD4* was overexpressed and the disruption was complemented. Therefore, all obtained strains were correct. The expression of *KmPFD4* in YSY005 was 5.15-fold higher than that in YSY007 without inhibitors. Whereas, with the presence of inhibitors, the expression of *KmPFD4* in YSY005 was 19.17-fold higher than that in YSY007.

### The effect of *KmPFD4* disruption or overexpression on inhibitor tolerance

During the pretreatment of lignocellulosic biomass, chemicals which inhibit cellulolytic enzyme and microbial growth and fermentation were produced. Different pretreatments produce different inhibitors in various ratios [6, 16]. Thus, it is difficult to set defined inhibitors composition in the inhibitor evaluation study. *K marxianus* is reported that it has higher tolerance to single kind inhibitor than *S. cerevisiae*. It completely assimilated furfural and vanillin in 8 and 16 h at an initial concentration of 2 g/L, respectively. Whereas, *S. cerevisiae* showed a lag assimilation period of 24 and 30 h, respectively [28]. In this study, a simplified inhibitors mixture was used to evaluate the effect of genetic modification on inhibitor tolerance. Furfural and HMF were selected as the furan compounds. Since formic and levulinic acids are degradation products of furan compounds [8], they were not added to the inhibitor mixture. Acetic acid was used as a weak acid. Because the pH of medium was adjusted to 6.0, the acetic acid existed as acetate. The phenolic compounds were more complicated, and 4-hydroxybenzaldehyde, syringaldehyde, catechol, and vanillin were included in the mixed inhibitors because they are the main phenols in lignocellulosic biomass hydrolysate [4, 14]. As the pretreated lignocellulosic biomass contains too many inhibitors, not all inhibitors were added to the medium to evaluate the yeast tolerance in many cases. Unrean et al. [11] added only acetate and furfural to the medium in their mechanistic analysis of inhibitor tolerance. Binary combinations of inhibitors were used in another inhibitor tolerance analysis [14]. Since inhibitors synergistically inhibit yeast growth and fermentation [14, 46], studies on tolerance of multiple inhibitors are more important. In this study, the mixture of inhibitors included three types of inhibitors which mimic the inhibitors in pretreated lignocellulosic biomass.

**Fig. 3:**
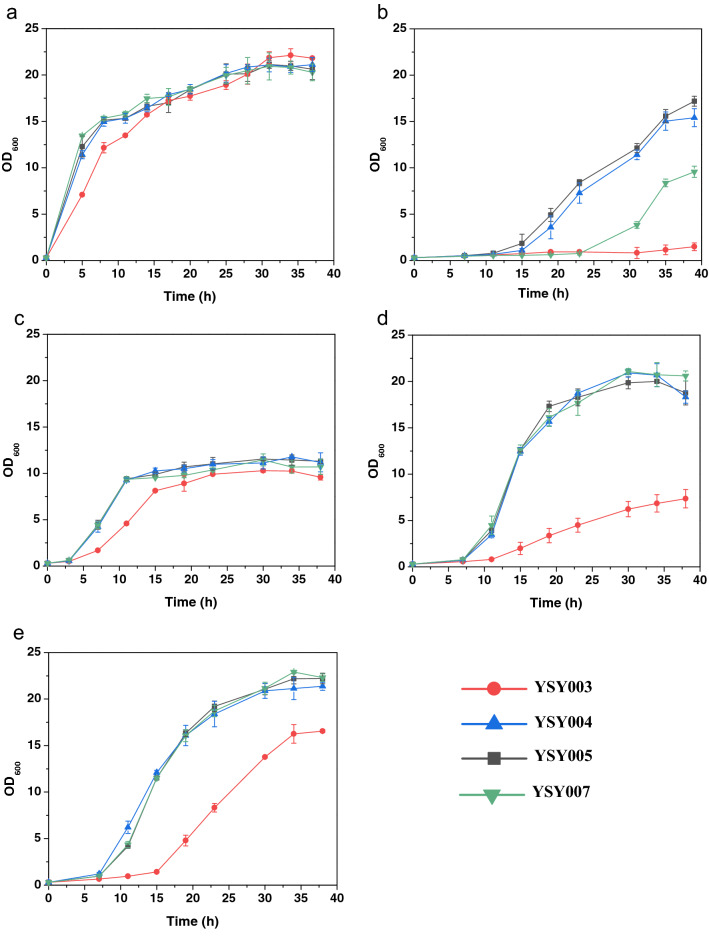
The effect of *KmPFD4* disruption or overexpression on tolerance to lignocellulosic biomass-derived inhibitors. YPD medium containing no inhibitor (**a**), mixed inhibitors (**b**), acetate sodium (**c**), furfurals (**d**), or phenols (**e**) was used to evaluate growth. All values are the means of three biological replicates ± standard deviation at each of the time points

Disruption of *KmPFD4* reduced the tolerance of *K. marxianus* to inhibitors. For fairly comparing the growth of each strain, YSY007 was used as control strain due to the same auxotroph (*leu2*) of YSY003, YS004, YSY005, and YSY007. Without inhibitors, the growth of *KmPFD4*-disrupted strain YSY003 was slightly weaker than that of the wild-type strain YSY007, and the biomass produced was similar (Fig. 3a). The final cell density (OD_600_) of each strain was approximately 20. The growth of YSY003 was apparently weaker than that of YSY007 when the medium contained inhibitors. When the medium contained mixed inhibitors, YSY003 did not grow, whereas YSY007 grew with a 24 h lag phase and the final cell density was only 9.57 (OD_600_) after 39 h of cultivation (Fig. 3b). When YSY003 was cultivated in a medium containing a single inhibitor, it had a longer lag phase and lower biomass production than YSY007 (Fig. 3c–e). The lag phases of YSY003 in the presence of acetate sodium (Fig. 3c), furfurals (Fig. 3d) and phenols (Fig. 3e) were 3, 12, and 15 h, respectively, whereas the lag phases of other strains were similar with each other, approximately 3, 7, and 7 h, respectively. The final cell densities of YSY003 in the presence of acetate sodium (Fig. 3c), furfurals (Fig. 3d) and phenols (Fig. 3e) were 10.28, 7.35, and 16.56 (OD_600_), respectively. The final cell densities of the other strains were also similar, approximately 11, 20, and 22 (OD_600_), respectively (Fig. 3c–e).

Overexpression of *KmPFD4* rescued the weak growth of YSY003 and enhanced the tolerance to mixed inhibitors. The growth of complementary strain YSY004 in the medium without inhibitors was similar to that of YSY007 (Fig. 3a) and stronger than YSY003 in the presence of a single inhibitor (acetic acid, furfurals, or phenols) (Fig. 3c–e). Furthermore, YSY004 grew better than YSY007 in the presence of mixed inhibitors with a 12 h lag phase and final cell density of 15.40 (OD_600_) (Fig. 3b).

The growth of the overexpression strain YSY005 indicated that overexpression of *KmPFD4* improved the tolerance to multiple inhibitors. YSY005 showed similar results to YSY004 in the medium with a single inhibitor and grew better than YSY004 with a final cell density of 17.19 (OD_600_) in the presence of multiple inhibitors (Fig. 3b).

**Fig. 4:**
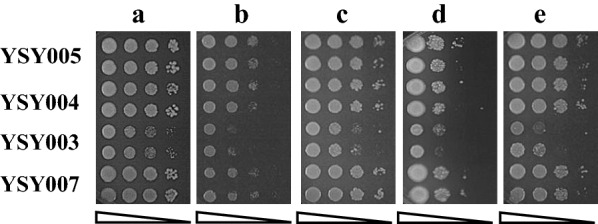
Spot assay of various strains on YPD plates containing no or various inhibitors. **a** No inhibitor, **b** mixed inhibitors, **c** acetic sodium, **d** furfurals, and **e** phenols. Two lines of each strain were determined. The concentrations of spotted cells were 6 × 10^–1^, 6 × 10^–2^, 6 × 10^–3^, and 6 × 10^–4^ (OD_600_), respectively

To show the effect directly, the effect on tolerance to inhibitors was also evaluated on the YPD plate containing various inhibitors (Fig. 4). The growth of YSY003 was slightly weaker than that of the other strains without the inhibitors (Fig. 4a), which was consistent with the results of liquid culture. In the presence of inhibitors, the growth of YSY003 was weaker than that of other strains, especially with the presence of mixed inhibitors or phenols, the growth decreased obviously. In the presence of furfurals, all strains grew worse than those on the plates without inhibitors, and the difference in growth between YSY003 and other strains was not obvious. These results were consistent with the growth curve results (Fig. 3c). Therefore, the disruption of *KmPFD4* may have less effect on the furfural tolerance of *K. marxianus.*

The overexpression of *KmPFD4* rescued the deficient growth of gene disruption on the plate containing inhibitors. However, the difference in growth among the overexpression strain YSY005, complementary strain YSY004, and control strain YSY007 was not obvious on solid medium (Fig. 4).

Very high or very low concentrations of inhibitors would eliminate the difference in tolerance (all would grow well or not at all) (data not shown). Therefore, the concentration of inhibitors used in this study was adjusted to reflect the difference in tolerance. Therefore, the concentration of single inhibitor was different to that in mixed inhibitors. It is not comparable between different single-type inhibitors. Since gene expression and metabolic regulation are different, the difference in tolerance to inhibitors in liquid medium or on solid plates can be expected.

It was not expected that overexpression of *KmPFD4* would enhance the tolerance to mixed inhibitors, whereas no enhancement was detected in tolerance to each type of inhibitor. Since multiple inhibitors can hinder microbial growth and fermentation synergistically [14, 46], the mixed inhibitors led to severe stress on yeast cells. It is possible that the native *KmPFD4* expression level is sufficient for a single type of inhibitor tolerance. Therefore, the overexpression of *KmPFD4* did not lead to increased tolerance to a single kind of inhibitors. In addition, *KmPFD4* was not effective for all inhibitors. Finally, the concentration of single inhibitor may not be high enough to make a difference of tolerance between overexpression and control strains. Therefore, overexpression of *KmPFD4* only showed apparent tolerance to mixed inhibitors. As a result, YSY003 did not grow with the presence of mixed inhibitors while other strains had a longer lag phase than with the presence of single type of inhibitor (Fig. 3b).

### The disruption of *KmPFD4* disturbed the cytoskeleton assembly

Actin and tubulin are the main components of the cytoskeleton, and cytoplasmic prefoldin is important for the folding of actin and tubulin monomers during cytoskeleton assembly [29]. Therefore, the effect of *KmPFD4* disruption on the cytoskeleton in *K. marxianus* was determined.

**Fig. 5:**
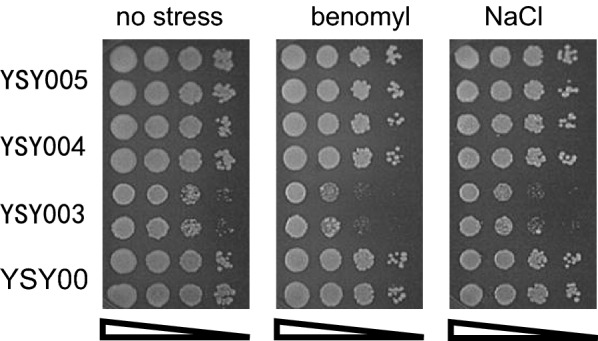
Growth of YSY003, YSY004, YSY005, and YSY007 with 7 μg/mL benomyl or 0.7 M NaCl. The concentrations of spotted cells were 6 × 10^–1^, 6 × 10^–2^, 6 × 10^–3^, and 6 × 10^–4^ (OD_600_), respectively

First, the effect of *KmPFD4* disruption on tubulin was evaluated. As shown in Fig. 5, the growth of YSY003 (*KmPFD4*-disrupted) was weaker than that of other strains on the plate containing benomyl or NaCl. These results indicated that the disruption of *KmPFD4* increased the sensitivity of the cells to benomyl, which interfered with tubulin assembly. In addition, disruption led to increased sensitivity of the cells to osmotic pressure (0.7 M NaCl). Disruption of *KmPFD4* may reduce the self-repair ability of cells to microtubule disorder, which is driven by inhibitors. In *S. cerevisiae*, *PFD4* disruption leads to increased sensitivity to benomyl, although the difference is not so apparent with NaCl [45]. This is possibly due to the different yeasts or the function of PFD4 in different yeasts.

**Fig. 6:**
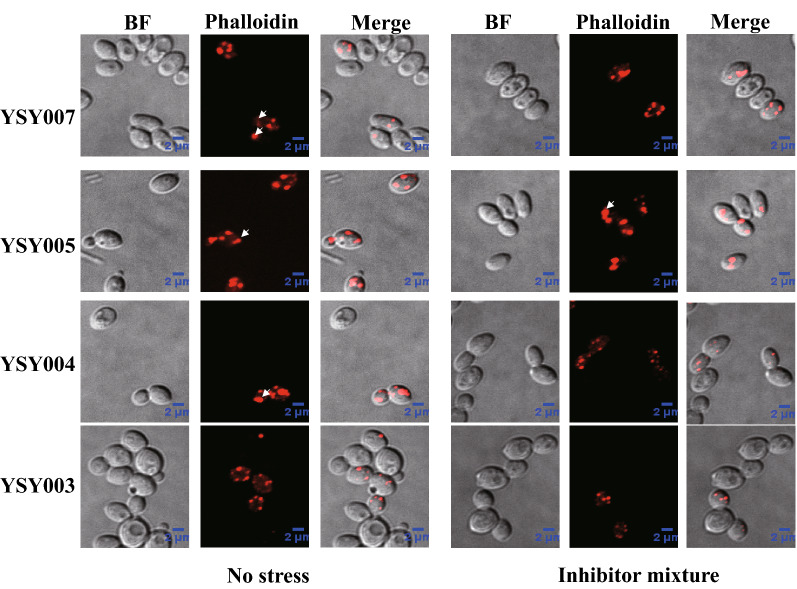
TRITC-phalloidin staining of YSY003, YSY004, YSY005, and YSY007 F-actin with or without the presence of mixed inhibitors. The white arrows indicate the patches or cables of F-actin

Second, the effect of *KmPFD4* disruption on actin was evaluated. As shown in Fig. 6, F-actin was stained with TRITC-phalloidin. Without inhibitors, the F-actins in most cells of YSY004, YSY005, and YSY007 existed as polarized patches (86.5%, 88.9%, and 86.9%, respectively), while polarized actins decreased (54.2%, 55.6%, and 47.7% respectively) in the presence of inhibitors (Fig. 6). In contrast, in the *KmPFD4*-disrupted strain YSY003, F-actin in most cells was depolarized even without inhibitors (23.2% polarized), and the ratio of the cells containing polarized actin decreased to 13.2% in the presence of inhibitors. It is possible that the disruption of *KmPFD4* interfered with the assembly of actin and led to depolarization (Fig. 6).

*KmPFD4* disruption also changed the cells shape of the *K. marxianus*. As shown in Fig. 6, the average length, width, and ratio of *KmPFD4*-disrupted cells (YSY003) were 4.00 μm, 3.47 μm and 1.17, whereas the length, width, and ratio of control cells (YSY007) were 4.01 μm, 2.46 μm and 1.65, respectively. Since the shape of cells was related to the cytoskeleton, these results also indicated that the disruption of *KmPFD4* interfered with the cytoskeleton assembly.

There are almost no reports connecting the tolerance of lignocellulosic biomass-derived inhibitors to prefoldin and the cytoskeleton. Consistent with the role of prefoldin in actin and tubulin folding, the deletion of prefoldin-encoding genes in *S. cerevisiae* results in impaired cytoskeleton functions, although none of the prefoldin subunits are essential for yeast viability [29]. In this study, the presence of inhibitors led to the disturbation of F-actin in the control strain, indicating that the cell skeleton is a possible target of inhibitors. In another study, the actin cytoskeleton was thought to be a cellular target for oxidative stress [47]. Lignocellulosic biomass-derived inhibitors suppress yeast growth and viability by producing stresses, such as ROS and membrane permeability. The accumulation of ROS leads to damage of the mitochondria and vacuole membranes, actin cytoskeleton, and nuclear chromatin [12, 48]. The prefoldins help the assembly of actin and tubulin, and the disruption of the prefoldin gene may reduce cytoskeleton assembly efficiency and reduce tolerance to stress. Therefore, the inhibitors disturbed the actin cytoskeleton structure and reduced cell viability. The disruption of *KmPFD4* led to disruption of the actin cytoskeleton assembly. Thus, the actin cytoskeleton would be destroyed further in the presence of inhibitors. As a result, the inhibitor tolerance ability decreased. Overexpression of *KmPFD4* may enhance the repair ability of the cytoskeleton through prefoldin and improve the inhibitor tolerance. In this study, the cytoskeleton disorder caused by disruption of *KmPFD4* is a possible reason for decreased inhibitor tolerance.

### The intracellular ROS concentration increased with the presence of inhibitors in the *KmPFD4*-disrupted strain

**Fig. 7:**
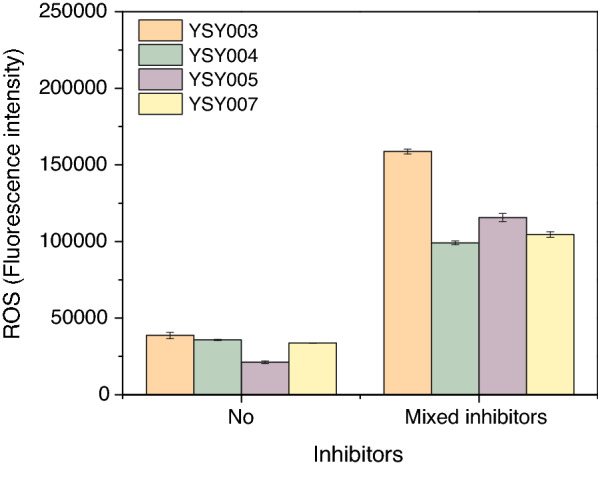
Disruption of *KmPFD4* led to greater ROS accumulation with the presence of mixed inhibitors. All values are the means of three biological replicates ± standard deviation at each of the time points

As lignocellulosic biomass-derived inhibitors produce ROS during their catabolism [12, 48], the overproduced ROS should be degraded to avoid harm to yeast cells. The ROS level of YSY003, YSY004, YSY005, and YSY007 with the presence of inhibitors were determined (Fig. 7). Without inhibitors, the intracellular level of ROS in each strain was similar, although the ROS level of YSY003 was slightly higher than that of the other strains. In the presence of mixed inhibitors, the ROS concentration of all strains increased, and the intracellular ROS level of YSY003 was 1.52-, 1.60-, 1.37- fold higher than that of the YSY007, YSY004, YSY005 (Fig. 7). It was unexpected that the ROS level of *KmPFD4* overexpression strain YSY005, which grow best with the presence of inhibitors (Fig. 3b) was not the lowest. Though we repeated the experiment, the results were similar. The reason is not clear. The *PFD4* (*GIM3*) disruption in *S. cerevisiae* led to greater sensitivity to H_2_O_2_ compared with the disruption of other prefoldin subunits; however, the disruption of *PFD2*, *5*, and *6* [45] did not change the sensitivity to H_2_O_2_. Therefore, it is possible that *KmPFD4* is important for the removal of superoxide or hyperoxide, which is a component of ROS. However, prefoldins are thought to be co-chaperones in protein folding, especially in cytoskeleton assembly. Why disruption of *KmPFD4* led to ROS accumulation was not clear. Therefore, subcellular localization and transcriptome analysis were conducted.

### KmPFD4 was localized in both the nucleus and cytoplasm in the presence of inhibitors

**Fig. 8:**
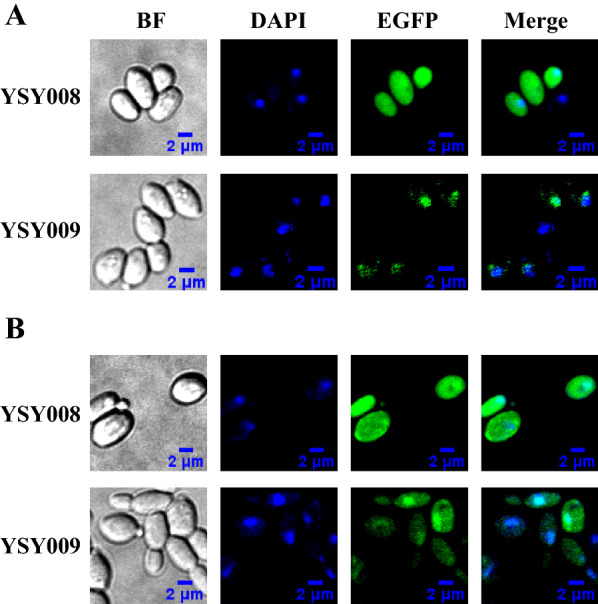
The intracellular localization of KmPFD4. **a** Without inhibitors, and **b** with mixed inhibitors. YSY008, expressing EGFP; YSY009, expressing KmPFD4-EGFP

After the effects of disruption and overexpression of *KmPFD4* on tolerance to inhibitors were evaluated, the intracellular location of PFD4 was determined to identify the possible mechanism of inhibitor tolerance. YSY008 was the control strain that only expressed EGFP, and YSY009 expressed the fusion protein KmPFD4-EGFP. Without inhibitors, EGFP was distributed in the cytoplasm of YSY008, whereas the green fluorescence of fusion protein in YSY009 cells was focused in or near the nucleus, which was stained with DAPI (blue) (Fig. 8a). These results indicated that KmPFD4 could enter the nucleus or accumulate around it. In the presence of inhibitors in the medium, KmPFD4 was distributed in both the nucleus and cytoplasm, although the fluorescence in the nucleus was stronger (Fig. 8b).

In eukaryotes, the prefoldins are localized to both the cytoplasm and nucleus [30]. In the cytoplasm, the prefoldins are mainly related to actin and tubulin assembly and in the nucleus, they regulate gene expression (reviewed by Laura Payán-Bravo [30]).

In this study, KmPFD4 was mostly localized near or within the nucleus, while in the presence of inhibitors, some KmPFD4 was transported to the cytoplasm. It is possible that the inhibitors disturbed the structure of the cytoskeleton, then the prefoldin including KmPFD4 moved to the cytoplasm to repair the damage. Furthermore, KmPFD4 may regulate gene expression due to its localization to the nucleus. However, the gene expression regulation function of *KmPFD4* has seldom been reported. Therefore, the transcriptome of the *KmPFD4*-disrupted strain was analyzed.

### Transcriptomic analysis of the *KmPFD4*-disrupted strain with multiple inhibitors indicated that *KmPFD4* regulated the gene expressions required for inhibitor tolerance

As KmPFD4 is located in or near the nucleus, the transcriptome was used to verify whether the disruption of *KmPFD4* changed the gene expression. We conducted transcriptomic analysis of *K. marxianus* YSY003 (*KmPFD4*-disrupted) and YSY007 (no disruption of *KmPFD4*) with or without lignocellulosic biomass-derived inhibitors by using RNA-seq. The levels of gene expression, normalized as TPM, were applied to the FCs of the DEGs (with absolute FCs ≥ 2; *p* < 0.001). The RNA-seq results were included in the following two relevant pairwise comparisons of gene expression levels: YSY007-I vs. YSY007-C (with vs. without inhibitors), and YSY003-I vs. YSY003-C (with vs. without inhibitors). Almost no read of *KmPFD4* was detected in YSY003 and this confirmed that *KmPFD4* was disrupted in YSY003 (Additional file 1).

Furthermore, the expression of 12 genes was analyzed by RT-PCR for validation of RNA seq results. With the presence of the inhibitors, the expression of mitochondrial peroxiredoxin (*PRX1*), superoxide dismutase [Mn] (*SOD2*), and succinate dehydrogenase [ubiquinone] (*SDH1*) was up-regulated in YSY007 and down-regulated in YSY003. Though Cu/Zn superoxide dismutase (*SOD1*), nicotinamide-nucleotide adenylyltransferase 1 (*NMNAT*), NADH pyrophosphatase (*NUDC*), and multicopy suppressor of SNF1 mutation (*MSN2*) were up-regulated in both YSY007 and YSY003, the changes in YSY003 was less than that in YSY007. Peroxisomal catalase A(*CTA1*) and citrate synthase (*CIT1*) were up-regulated in YSY007, and down-regulated in YSY003. ATP synthase subunit 4 (*ATP4*) was down-regulated in both YSY003 and YSY007. Glucose-6-phosphate isomerase (PGI1) and glyceraldehyde-3-phosphate dehydrogenase 3 (*TDH3*) were up-regulated in both YSY003 and YSY007. Though the expression changes of the analyzed genes were not the same as RNA-seq results, their trends of the changes in real-time PCR were similar to those in RNA-seq (Additional file 2: Table S2).

Based on these results, we focused on the DEGs in *K. marxianus* YSY007 or YSY003 with or without the multiple inhibitors (I vs. C), especially those DEGs related to ROS detoxification, central carbon metabolism, mitochondrial respiratory chain, NAD(P)^+^ biosynthetic enzymes, and nicotinate metabolism. There were some changes of the gene expression with the presence of inhibitors when *KmPFD4* was disrupted. Though it was still difficult to make a conclusion that KmPFD4 regulated the gene expression, KmPFD4 did affect the gene expression directly or indirectly.

The genes related to ROS detoxification were not upregulated in the *KmPFD4*-disrupted strain in the presence of inhibitors. Since ROS is produced during the metabolism of inhibitors [12], removing over-accumulated ROS is important for inhibitor tolerance. As expected, when exposed to the stress of multiple inhibitors, most of the DEGs related to ROS detoxification were upregulated, including *SOD1, SOD2, PRX1, GPX2,* peroxiredoxin (*HYR1*)*,* thioredoxin reductase(*TRR1*)*,* glutamate–cysteine ligase (*GSH1*)*,* superoxide dismutase 1 copper chaperone (*CCS1*)*,* glutaredoxin-1 (*GRX1*)*,* monothiol glutaredoxin-5 (*GRX5*)*,* peptide methionine sulfoxide reductase *(PMSR*)*,* NADPH-dependent methylglyoxal reductase (*GRE2*)*,* thioredoxin-2 (*TRX2*), thioredoxin-3 (*TRX3*)*,* and putative uncharacterized oxidoreductase gene (*WCAG*), while cytosolic catalase T (*CTT1*)*,* peroxiredoxin (*DOT5*)*, glutathione S-transferase 1* (*GST1*)*,* and Cys-Gly metallodipeptidase (*DUG1*) were downregulated in the YSY007-I vs. YSY007-C group, as previously reported [27]. However, in the *KmPFD4*-disrupted YSY003-I vs. YSY003-C group, although the expression of *GPX2*, *HYR1*, *TRR1*, *TRX1*, and *GRE2* were upregulated as in the YSY007-C vs. YSY007-I group, most genes were not significantly regulated in the YSY003-C vs. YSY003-I group (Additional file 1). These results indicated that the expression of genes involved in ROS elimination in the *KmPFD4* disruption strain was relatively unchanged compared to the wild-type strain in response to the presence of inhibitors.

The disruption of *KmPFD4* reduced the expression of the genes of the respiratory chain and hindered ATP generation. The mitochondrial respiratory chain forms membrane potential to produce ATP and transfers electrons in the form of multi-enzyme complexes. As shown in the additional file 1, under the stress of the multiple inhibitors, rotenone-insensitive NADH-ubiquinone oxidoreductase (*NDI1*)*,* external NADH-ubiquinone oxidoreductase 1 (*NDH1*)*, SDH1/2/3/4,* and ubiquinol cytochrome-c reductase complex (*QCR9*) were upregulated and there was no significant change in *QCR1,* cytochrome b-c1 complex subunit Rieske (*RIP1*)*, QCR6*, and *ATP4* in the control (YSY007-I vs YSY007-C) pairwise comparison. Whereas in the YSY003-I vs. YSY003-C group, there was no significant change in *NDI1* or *SDH1/2/3/4*. Furthermore, *QCR1, RIP1, QCR6, QCR9*, and *ATP4* were downregulated. The expression of the counterpart genes in the YSY003-I vs. YSY003-C group was downregulated compared with those in the YSY007-I vs. YSY007-C group. These results suggested that with the stress of multiple inhibitors, disruption of *KmPFD4* reduced the expression of the genes of the respiratory chain and may hinder ATP generation further. In previous studies, in the presence of inhibitors, the expression of genes related to ATP production was upregulated due to increased ATP required for inhibitors degradation and removal [26, 27]. However, in the wild-type strain, the intracellular concentration of ATP is decreased due to increased consumption [49], disruption of the proton gradient of mitochondria [50], non-specific hydrolysis of ATP [49], and decreased intracellular concentration of NADH for ATP production. Decreased genes expression in the respiratory chain led to more severe shortage of ATP. ATP is required to pump the proton in the presence of a weak acid [11]. In addition, ATP is the adenylyl-backbone donor for NAD^+^. The shortage of ATP is expected to reduce inhibitor tolerance.

The genes related to NAD^+^ biosynthetic enzymes and nicotinate metabolism were not upregulated in the *KmPFD4*-disrupted strain compared to the control strain. The essential coenzymes nicotinamide adenine dinucleotides, NAD(P)^+^ and NAD(P)H, participate in key redox reactions and contribute to maintaining cells fitness and genome stability [51]. NAD^+^ is important in lignocellulosic biomass-derived inhibitor tolerance and is the source of total NAD(P)^+^ and NAD(P)H [26]. Under the stress of the multiple inhibitors, almost all of the DEGs related to NAD^+^ biosynthetic enzymes and related proteins were upregulated, except *PNC1* encoding nicotinamidase in the YSY007-I vs. YSY007-C group. However, in the YSY003-I vs. YSY003-C group, these genes were not significantly regulated except for nucleoside transporter *FUN26* which was downregulated. This may imply that the NAD^+^ biosynthesis in the *KmPFD4*-disrupted strain was not enhanced as those in the wild-type strain in response to the presence of inhibitors. Therefore, the supply of NAD^+^ in the *KmPFD4*-disrupted strain was not improved with the increased demand for inhibitor tolerance, and the tolerance ability was decreased.

In the presence of inhibitors, the genes related to glycolysis were up-regulated, in the *KmPFD4*-disrupted strain. In contrast, most of the genes related to glycolysis were down-regulated or no significant change in the non-disrupted strain which was also reported in *S. cerevisiae* [9]. Central carbon metabolism plays an important role in the carbon source and energy production in yeast cells. As shown in Additional File 1 s, in the presence of multiple inhibitors, quite a few DEGs related to glycolysis/gluconeogenesis, such as *PGI1, 6-*phosphofructokinase subunit (*PFK1/2*)*,* triosephosphate isomerase (*TPI*)*, TDH3,* phosphoglycerate kinase (*PGK*)*,* enolase (*ENO*)*,* pyruvate kinase (*PYK*)*,* Pyruvate decarboxylase (*PDC*) etc., were upregulated in the YSY003-I vs. YSY003-C group, while there was no significant change in YSY007-I vs. YSY007-C pairwise comparison.

On the other hand, among those DEGs in the TCA cycle, *SDH1/2/3/4* and malate dehydrogenase (*MDH1/2*) were upregulated and there was no significant change to *IDP2* in YSY007-I vs. YSY007-C pairwise comparison. On the other hand, in the YSY003-I vs. YSY003-C group, there was no significant change in the expression level of *SDH1/2/3/4* and *MDH1*. Furthermore, *MDH2* and *IDP2* were downregulated. Likewise, isocitrate lyase (*ICL1*) was downregulated in YSY003-I vs. YSY003-C pairwise comparison, but there was no significant change in YSY007-I vs. YSY007-C pairwise comparison (Additional file 1). These results indicated that the disruption of *KmPFD4* stimulated glycolysis/gluconeogenesis, but hindered the TCA cycle. It is well-known that the TCA cycle provides more ATP and NAD(P)H, which are necessary for inhibitors removal and conversion. Our previous study have shown that the intracellular concentrations of ATP and NAD(P)H decreased due to the consumption of inhibitors [26]. The disruption of *KmPFD4* led to a severe shortage of ATP and NAD(P)H and finally reduced the inhibitor tolerance.

The results of the TTC staining indicated that the disruption of *KmPFD4* decreased the respiration. On the plates, the YSY007 colonies were red, whereas, the color of YSY003 colonies was lighter than that of YSY007. And the white colonies indicated much weaker respiration. These results illustrated that the respiration of *K. marxianus* was interfered after *KmPFD4* disruption (Additional file 2: Fig S4).

The expression of genes related to the *MSN2/4*mediated stress response element (STRE) is intricate. Transcriptional activator MSN2 in *S. cerevisiae* regulates the transcription of the genes associated with oxidative stress, heat shock, and high concentration of ethanol [52]. Because furfural and phenolic inhibitors lead to intracellular ROS accumulation [12, 13], the expression of MSN2/4 mediated stress response elements in *K. marxianus* were also analyzed through RNA-seq. *MSN4* was not found in the genome of *K. marxianus*, and the *KmMSN2* expression was downregulated in YSY003 with the presence of inhibitors, whereas in YSY007, its expression was upregulated (Additional file 1). Though the expression levels of several genes such as hexokinase 1 (*HXK1*)*,* glycogen phosphorylase (*GPH1*), heat shock protein *SSA3,* aldehyde dehydrogenase 5 (*ALD5*)*,* heat shock protein 26 (*HSP26*) and heat shock protein 31 (*HSP31*) etc. were similar in both pairwise comparisons, most regulations of DEGs were different between the pairwise comparisons, and these genes also presented in ROS or central carbon metabolism groups (Additional file 1). The expression of *SSA3*, *SOD2*, *TDH2*, *HSP26*, *HSP31*, *MDH1*, and *MDH2* which enhance the stress tolerance was upregulated with the presence of inhibitors in YSY007. The furfural tolerance of *S. cerevisiae* is improved through overexpression of *MSN2*. Various kinds of antioxidant enzymes are highly expressed by the constitutive overexpression of *MSN2* even under the non-stress condition, therefore, yeast cells adapt to exposure to acute oxidative stress caused by furfural [52]. In this study, the upregulated expression of *MSN2* and some STRE genes in YSY007 with the presence of inhibitors indicated increased response to inhibitors and the ROS produced by these inhibitors. However, *KmPFD4* disruption led to decreased response to inhibitors.

Prefoldins can regulate gene expression not only as free single subunit but also as a prefoldin complex. Canonical prefoldin interacts with specific transcription factors and modulates their activity [30]. The PFD1 [31], PFD3 [32] [53], PFD5 [54], PFDN1-PFDN2-PFDN5- PFDN6 complex [36], and PFD5-PFD6 complex [35] were reported that they can regulate gene expression in mammalian cells, hepatitis B, yeast, or plants. However, there have been few reports regarding PFD4 in the regulation of gene expression. Iijima et al. reported that PFD4 (C-1 protein) is with possible transcription factor activity at the G(1)-S phase transition in human fibroblast cell lines [33], but no further study is reported. In S. *cerevisiae*, the absence of prefoldin subunits, but not the prefoldin complex, alters stress-induced transcription [45]. Amorim et al*.* reported that *PFD4* deletion in *S. cerevisiae* altered *TRX2*, *CTT1*, and *HSP26* transcription when cells were exposed to H_2_O_2_ [45]. The function of *KmPFD4* in the regulation of the genes involved in the tolerance of *K. marxianus* has not been reported yet.

The transcriptome analysis results indicated that the disruption of *KmPFD4* down regulated the expression of the most genes related to ATP and NAD(P)H synthesis, and the TCA cycle (Additional File 1). The expression of these genes did not respond to the presence of inhibitors after *KmPFD4* was disrupted. Therefore, a greater shortage of ATP or NAD(P)H occurred, and led to decreased detoxication ability to inhibitors and reduced ROS elimination ability. Thus, the inhibitor tolerance of yeast cells was decreased.

### The disruption of ***KmPFD4*** reduced the tolerance of ***K. marxianus*** to other stresses

**Fig. 9:**
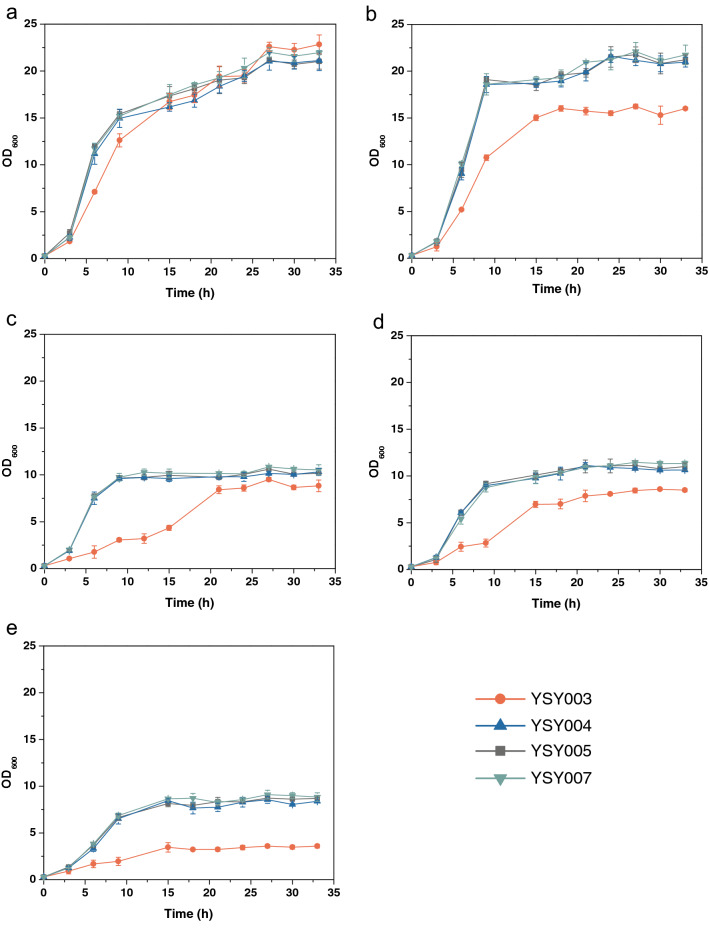
Disruption of *KmPFD4* reduced the tolerance of *K. marxianus* to various stresses. **a** No stress, **b** 180 g/L glucose, **c** 0.7 M NaCl, **d** 20 g/L ethanol, and **e** 45 °C. All values are the means of three biological replicates ± standard deviation at each of the time points

After the effect of *KmPFD4* disruption on the tolerance of *K. marxianus* to lignocellulosic biomass-derived inhibitors was determined, its effect on ethanol, temperature, and osmotic stress was evaluated by measuring their growth (Fig. 9). Therefore, the disruption of *KmPFD4* not only reduced the inhibitor tolerance of *K. marxianus,* but also reduced their tolerance to other stresses.

Microtubules and tubulin are important for cell survival and proliferation in glucose-starved non-small cell lung cancer cells [55], chemotherapy-resistant tumor cells [56] and mammalian cells exposed to H_2_O_2_ stress [57]. They are also correlated with arsenic resistance in *S. cerevisiae* [58], the response to heat stress in *S. cerevisiae* [59] and hydrostatic pressure tolerance in *Schizosaccharomyces pombe* [60]. Increase of external osmolarity caused loss of actin filament cable redistribution of cortical actin filament patches [61]. The actin cytoskeleton is a cellular target for oxidative stress [47]. High concentration of ethanol impaired cellular wall permeability by disrupting sorting and signaling functions and provoking an increase in cell size, which caused a cell cycle delay. This correlates with the disappearance of actin cables and the diffusion of actin patches in yeast cells [62, 63]. Since *KmPFD4* is one of the units of prefoldin that assists the assembly of the cytoskeleton, it is expected that *KmPFD4* is important in stress other than lignocellulosic biomass-derived inhibitors. However, the overexpression of *KmPFD4* did not improve tolerance to these stresses. It is possible that osmotic, ethanol, and temperature stresses are not strongly related to ROS. Thus, the function of *KmPFD4* in the expression of genes related to ROS was not reflected.

### Overexpression of ***KmPFD4*** improved the fermentation ability of ***K. marxianus***

**Fig. 10:**
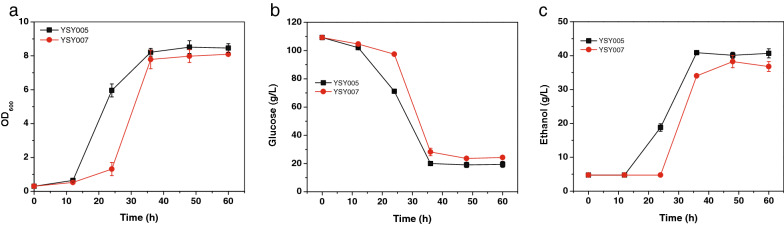
Anaerobic fermentation with mixed inhibitors using *KmPFD4* overexpression strain YSY005 and control strain YSY007. **a** Growth, **b** glucose consumption, and **c** ethanol production. All values are the means of three biological replicates ± standard deviation at each of the time points

As overexpression of *KmPFD4* improved the tolerance of *K. marxianus* to mixed inhibitors, ethanol fermentation from glucose in the presence of mixed inhibitors was also determined. From 100 g/L glucose, YSY005 produced 35.11 g/L ethanol in 36 h with a productivity of 0.98 g/(L·h), whereas YSY007 only produced 32.23 g/L ethanol in 48 h with a productivity of 0.67 g/(L·h) (Fig. 10). Ethanol production of YSY005 increased only 8.93% (P value = 0.082), whereas, productivity of ethanol improved by 46.27% (P value = 0.022). These results indicated that overexpression of *KmPFD4* increased ethanol fermentation ability, especially in productivity with the presence of inhibitors. Since overexpression of *KmPFD4* did not improve ethanol tolerance obviously (Fig. 9d), it is possible that increased tolerance to inhibitors led to better growth, resulting in high ethanol productivity. The ethanol yield of YSY005 and YSY007 were 0.40 g/g and 0.38 g/g of consumed glucose, respectively. It is about 78.27% and 74.36% of theoretical yield (0.511 g/g). Overexpression of *KmPFD4* only improved 6.83% of the yield (P value = 0.048). These results were consistent with other reports that the inhibitors delayed yeast fermentation and did not significantly reduce the ethanol yield [64, 65]. Therefore, the improvement in ethanol production and yield was not as obvious as that in productivity through the overexpression of *KmPFD4*.

## Conclusions

*KmPFD4* affects the tolerance of *K. marxianus* to lignocellulosic biomass-derived inhibitors by maintaining the structure of the cytoskeleton and regulating the genes expression in response to ROS elimination and inhibitors degradation. Disruption of *KmPFD4* led to a more severely disordered cytoskeleton and reduced the viability of the cells in the presence of inhibitors. Furthermore, the disruption of *KmPFD4* led to a decrease in genes expression in response to the presence of inhibitors though the mechanism of KmPFD4 affecting gene expression needs further study. Thus, the supply of ATP and NAD(P)H required for inhibitor tolerance is decreased. Overexpression of *KmPFD4* enhanced the restoration of the disturbed cytoskeleton and improved inhibitor tolerance. Therefore, *KmPFD4* affects the tolerance to inhibitors in improving the cytoskeleton assembly and related gene expression. The study on *KmPFD4* is related to a wide spectrum of stress tolerance, which provides a potential route to improve the tolerance of yeast to multiple lignocellulosic biomass-derived inhibitors.

## Supplementary Information

**Additional file 1.** Functional categories and fold changes of comparative expression (in form of log_2_FC) of differentially expressed genes in tolerance to multiple inhibitors.

**Additional file 2.** Additional figures and tables.

## Data Availability

The dataset supporting the conclusions of this article are included within the article and its additional files.
