# Supplementary material for: Influence of prefoldin subunit 4 on the tolerance of Kluyveromyces marxianus to lignocellulosic biomass-derived inhibitors
Source: Microb Cell Fact. 2021 Dec 14;20:224. doi: 10.1186/s12934-021-01715-y (PMC8672639; doi:10.1186/s12934-021-01715-y)
Supplement: Supplementary file 2 — Additional file 2. Additional figures and tables. [file 12934_2021_1715_MOESM2_ESM.docx]

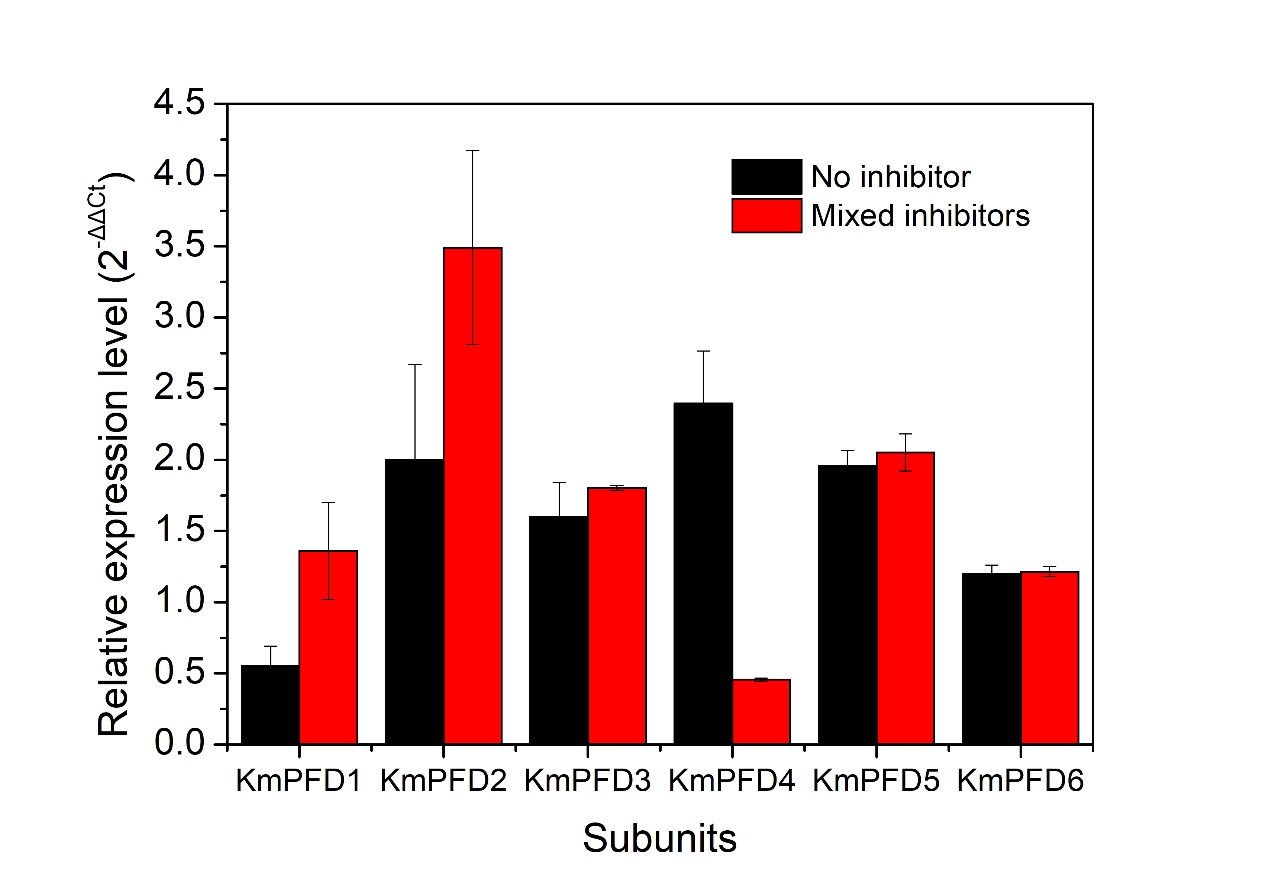


**Fig S1 Real-time PCR analysis of the *K. marxianus* prefoldin subunits expression at stationary phase *vs* exponential phase with or without inhibitors.**


390 bp

600 bp

600 bp

*KmPFD4* ORF

B.
 * * * * * *
KmPFD4 : MELLPEGQKNS-VTVAYEDQQRINEFSKLIMRKDAIDQELTQQRTEKEYLDDVSLEIELIDEDEKVQYKVGDVFVYLKQKEVVDKLTKDAS : 90
KlPFD4 : MELLPEGQKNT-VTVAYEDQQRINEFSKLIMRKDAIEQDLTQQRTEKEYLDDISLEIELIDEDEKVQYKIGDVFVFLKQKEVVEKLESDAE : 90
ScPFD4 : MELLPQGQRNN-TQVTFEDQQKINEFSKLIMRKDAIAQELSLQREEKEYLDDVSLEIELIDEDEPVQYKVGDLFIFMKQSKVTAQLEKDAE : 90
CaPFD4 : MELLPSGQANTSTEVTYEDQLKINKFSTLISKKDEQTQQLSTLKTEKEYLDDLSIELELIDDDEKIQYKVGDCFVFLPKDQVLEKIESDTD : 91


KmPFD4 : RIDEAIEKLENDEAELSSRIKDLKSILYAKFGDNINLER : 129
KLPFD4 : KIDSNIEKLESDEAELDSRIKELKSILYAKFGDNINLER : 129
ScPFD4 : RLDNKIETLEDKQRDIDSRLDALKAILYAKFGDNINLER : 129
CaPFD4 : SLEEKINSIEELIDGFDEELKDLKAQLYDKFGDNINLER : 130

**Fig. S2 The cloned *KmPFD4*** **and amino acid sequence alignment of PFD4. A.** Schematic of cloned *KmPFD4* gene**; B.** Alignment of amino acid sequences of KmPFD4 and homologous proteins in other species. Prefoldin subunit interaction sites [polypeptide binding] were shown with *. KmPFD4: PFD4 from *K. marxianus*, genbank accession No. BAP73153; KlPFD4: PFD4 from *K. lactis*, genbank accession No. QEU60778; ScPFD4: PFD4 from *S. cerevisiae*, genbank accession No. NP_014246; CaPFD4, PFD4 from *Candida albicans*, genbank accession No. KAF6071592.


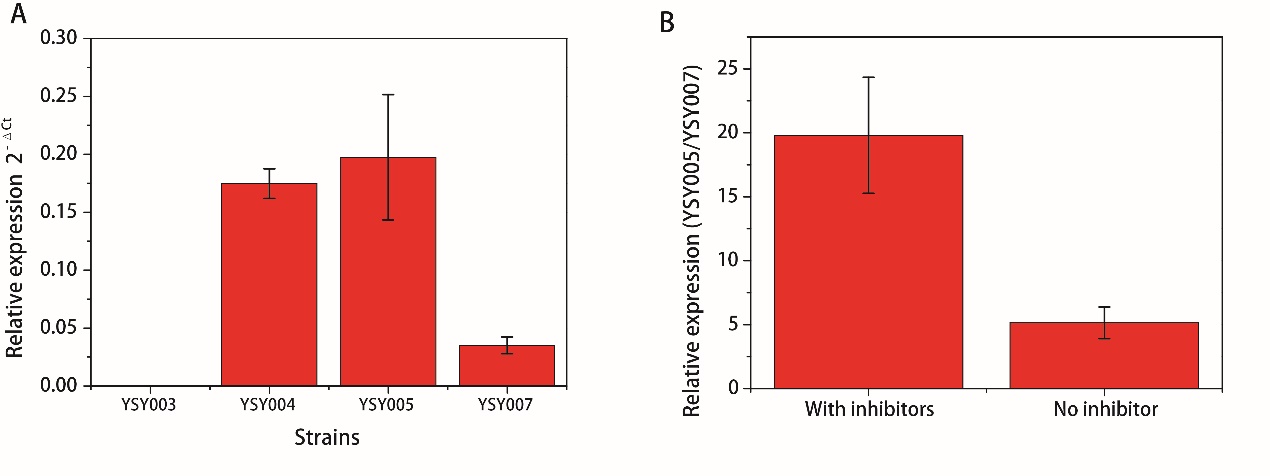


**Fig. S3 Real time PCR analysis of the *KmPFD4* expression level in constructed strain.** A. expression of KmPFD4 in each strain, B. The increased expression of KmPFD4 (YSY005 *vs* YSY007) with or without inhibitor. YSY003: *KmPFD4* disrupted, YSY004: complementary strain, YSY005: overexpression strain, YSY007: *KmPFD4* non-disrupted with same auxotrophic type as other strains (Control).

**
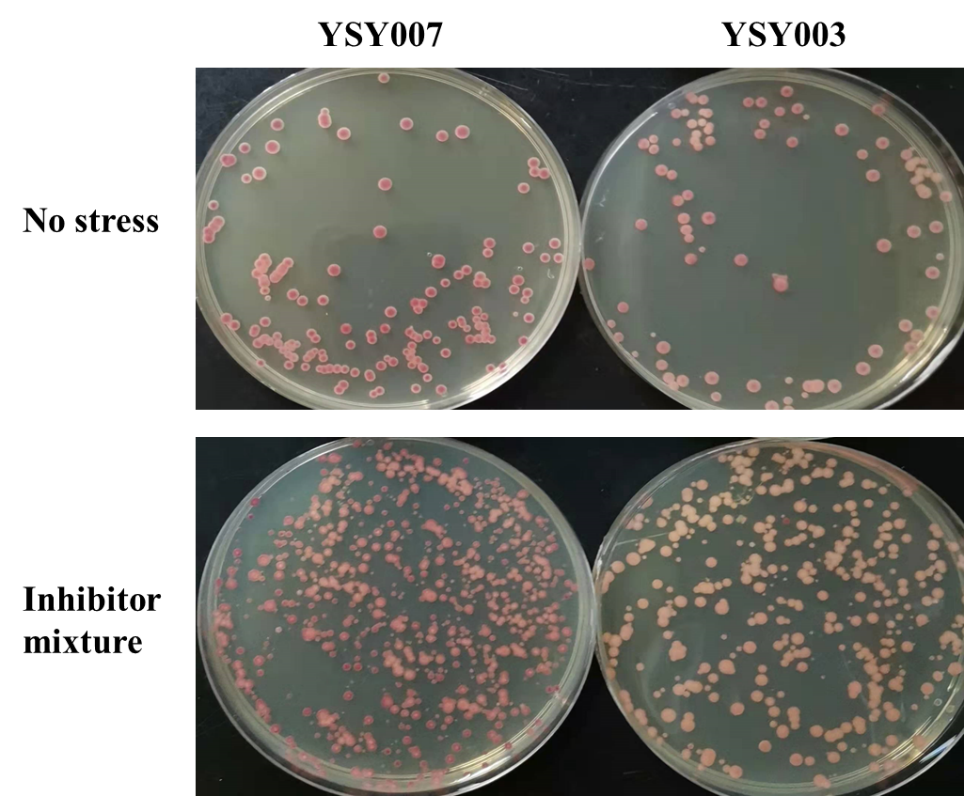
**

**Fig. S4 2,3,5-Triphenyltetrazolium chloride stain results.**

Δ

**Table S1. Primers used in this study.**

| Primer | Sequence (5’🡪 3’)* |
| --- | --- |
| KmPFD4-F  KmPFD4-R  KmPFD4-F2  KmPFD4-R2  ScURA3-SmaI-F | AGTGAATATAGACAAGCTTAACGTT  CGCACCCAAGGAATATAACGAAATA  TGCAACGGTAACAGAGTTCTTTTGG  ATTTTGTACGCCAAATTCGGCGACA  TCCCCCGGGTATTTAGAAAAATAAACAAATA |
| ScURA3-SmaI-R | TCCCCCGGGAATGCGTACTTATATGCGTC |
| ScURA3-F1 | ATGTCGAAAGCTACATATAAG |
| ScURA3-R2 | TTAGTTTTGCTGGCCGCATC |
| EGFP-EcoRI-F | CCGGAATTCCGGATGGTGAGCAAGGGCGA |
| EGFP-NotI-R | TTGCGGCCGCAATTACTTGTACAGCTCG |
| KmPFD4-EcoRI-F | CCGGAATTCATGGAGCTACTACCGGAAGGCCAAA |
| KmPFD4-NotI-R | ATTTGCGGCCGCTTAGCGCTCTAGGTTGATGTTGTCG |
| EGFP-fusion-F | CCTAGAGCGCGGTTCAGGTAGTGGTAGTGGATCTGTGAGCAAGG |
| KmPFD4-fusion-R | ACTACCACTACCTGAACCGCGCTCTAGGTTGATGTTGTCGCCGA |
| TDH3-Ter-R | CATTTTCGATTCATTGATTGA |
| RT-SOD1-F | TTACCCAAGTCACCAACA |
| RT-SOD1-R | AGAATCTGAAGACCAATCCA |
| RT-SOD2-F | GGTGTCTTGGTTGTAGGT |
| RT-SOD2-R | CACTGCTTGTTCTGGAAG |
| RT-PRX1-F | TCAGACTCATCATCACCTAC |
| RT-PRX1-R | ACAACGCCCTTAGAATCA |
| RT-CTA1-F | CTGATGTTACTGGCAAGTC |
| RT-CTA1-R | AATTCAACAGCACCAAGAG |
| RT-SDH1-F | CTTGTTGACTTGTGCTACTC |
| RT-SDH1-R | AGTGTGCTTCATCCAGTTA |
| RT-ATP4-F | GAACTCCTCCAGAACCAA |
| RT-ATP4-R | TTGTAATTGACGGACAGAAG |
| RT-NMNAT-F | ATGTCCGTTCGTTCTTATTG |
| RT-NMNAT-R | CGTGTTCTTGGATGTATCTG |
| RT- NUDC -F | ATGCTTCTGAGACGAGTT |
| RT- NUDC -R | TGATTGGATGCCTTGGTA |
| RT-MSN2-F | GCTCAACAGGCTATAATTCAG |
| RT-MSN2-R | CTTGCTACATTGGTCACATT |
| RT-PGI1-F | TGATTGCTTACTACGAACAC |
| RT-PGI1-R | ATCTTGCTACCATCATCCA |
| RT-TDH3-F | CAGCGTCTTCAGTGTAAC |
| RT-TDH3-R | GGTAAGGTCTTGCCAGAA |
| RT-CIT1-F | CTCCACCATCTACGAAGTT |
| RT-CIT1-R | CTCTTGAGACACCGAACA |
| RT-KmPFD1-F | AGTAATCAACCGCAGGAT |
| RT-KmPFD1-R | TTCGTCTAGCAACTTCTCA |
| RT-KmPFD2-F | ATTCTTGAGCAGTGGTAATC |
| RT-KmPFD2-R | AGGTCGTGATAAGGATGAA |
| RT-KmPFD3-F | AGATGTGATGCTTGAATACC |
| RT-KmPFD3-R | CATTTGCCTCCTTTGAACA |
| RT-KmPFD4-F | CAAATACATCGCCAACCTT |
| RT-KmPFD4-R | ATCAGCAACGCATCAATG |
| RT-KmPFD5-F | TTGATGGCTGCTACTCTC |
| RT-KmPFD5-R | TTGGTACTGGCTACTATGTC |
| RT-KmPFD6-F | GGTGTGCTACTTCCTGTT |
| RT-KmPFD6-R | CTACTGCTGTTGTTGTTGG |
| RT-KmACT1-F | TTGGCTGGTAGAGACATCACTGAC |
| RT-KmACT1-R | AGCAGATGATTGAGAAGCGGTTTG |

*Restriction enzymatic sites are underlined.

Table S2 confirmation of the RNA-seq results through real-time PCR*

| Locus tag | Genes | YSY003 | YSY007 |
| --- | --- | --- | --- |
| KMAR_70075 | *SOD1* | 1.03±0.17 | 1.91±0.07 |
| KMAR_20527 | *SOD2* | 0.76±0.17 | 3.56±1.14 |
| KMAR_60391 | *CTA1* | 0.62±0 | 5.08±0.25 |
| *KMAR_20443* | *SDH1* | 0.70±0.05 | 1.28±0.05 |
| *KMAR_10737* | *ATP4* | 0.70±0.07 | 0.92±0.06 |
| *KMAR_40052* | *NMNAT* | 1.06±0.05 | 4.99±0.28 |
| *KMAR_10620* | *NUDC* | 1.16±0.1 | 3.74±0.06 |
| *KMAR_40319* | *MSN2* | 1.00±0.02 | 2.00±0.13 |
| *KMAR_10734* | *PGI1* | 4.96±0.66 | 1.77±0.07 |
| *KMAR_80062* | *TDH3* | 10.21±1.73 | 1.85±0.15 |
| *KMAR_80342* | *PRX1* | 0.90±0.02 | 5.10±0.3 |
| *KMAR_20100* | *CIT1* | 0.87±0.13 | 22.18±2.07 |

The relative expression level is shown in 2^-ΔΔCt^
